# Health at the heart of climate action: Urgency of scaling adaptation globally

**DOI:** 10.1590/0037-8682-0249-2025

**Published:** 2025-09-22

**Authors:** Ethel Leonor Noia Maciel

**Affiliations:** 1 Universidade Federal do Espírito Santo, Vitória, ES, Brasil.; 2 Rede Brasileira de Pesquisa em Tuberculose, Rio de Janeiro, RJ, Brasil.

The escalating climate crisis poses unprecedented challenges to health systems, particularly in countries facing deep social and environmental inequalities, such as Brazil. The shifting patterns of infectious and chronic diseases, the increasing frequency of extreme weather events, food and water insecurity, and emerging environmental risks have disproportionately impacted historically marginalized populations, including Black, Indigenous, riverside, and peripheral urban communities^1^.

As Brazil’s Special Envoy for the Health and Climate Agenda at the 30th Conference of the Parties of the United Nations Framework Convention on Climate Change, I present not only a proposal in progress but also a collective commitment: the Belém Health Action Plan. Under consultation, it serves as a concrete response to Ambassador André Corrêa do Lago’s call for a global joint effort toward effective and monitoring solutions to the climate crisis.

More than being a technical document, the plan is designed to be practical, adaptable, and actionable, allowing countries to tailor it to their contexts, engage in collaborative actions, and view themselves within its framework. Its strength lies in its potential to establish a shared reference for monitoring, technical cooperation, and realistic financing strategies^2^.

We must recognize that the cost of inaction on public health is being paid through preventable illnesses, premature deaths, deteriorating quality of life, and unsustainable public health spending^3^. However, these costs remain largely invisible in the climate negotiations and resource allocation processes.

According to a recent Lancet Countdown report, global heat-related mortality among individuals over 65 years of age increased by 68% between 2012 and 2021 compared with that during 2000-2009^4^. Simultaneously, climate-sensitive diseases, such as dengue, malaria, and leptospirosis, have expanded their geographic reach, exposing millions to new risks^5^.

Therefore, health adaptation strategies must be anchored in clear evidence-based financial estimates for both immediate and long-term interventions. Countries must answer the following key questions: What will it cost to create climate-proof health systems over the next 5, 10, or 20 years? Which areas require urgent investment? What technologies and capacities must be deployed and when?

The answers to these questions are required to ensure that health is embedded in National Adaptation Plans, Nationally Determined Contributions, and the eligibility criteria for international climate financing^6^. Without them, the health sector will remain underfunded and reactive, instead of delivering proactive, coordinated, and scaled responses to these crisis demands.

The Belém Health Action Plan seeks to bridge these gaps by equipping countries to assess vulnerabilities and risks, plan and prioritize locally driven interventions, estimate costs and timelines, integrate health into global climate finance frameworks, and monitor implementation over time.

In Brazil, home to the world’s largest public health system, funding adaptation requires parliamentary engagement. Future national budgets must reflect the increasing health impacts of climate change. Historically, investments in health have focused on infrastructure. They must prioritize prevention and preparedness for climate-sensitive threats.

Lawmakers can play a pivotal role in this process. Parliaments can facilitate long-term planning and ensure the continuity of health investments by aligning legislative priorities with climate adaptation needs. Moreover, they help bridge national realities through global negotiations, fostering an alignment between local needs and international support mechanisms^7^.

Scientific communities are equally essential, as researchers can provide a technical foundation for identifying vulnerabilities, estimating their impact, and assessing the cost-effectiveness of adaptation measures. A strong science-policy interface is crucial for aligning national health strategies with climate risk projections and resource flows^8^.

To place public health at the heart of the climate agenda, it must be made visible through climate finance flows, adaptation funds, and planning instruments. The absence of dedicated health parameters in most multilateral climate frameworks hinders access to vital resources and weakens countries’ abilities to protect their most vulnerable populations^9^.

We cannot afford to wait, as the crisis is now. Responses must be coordinated, global, and grounded in justice. The Belém Health Action Plan is Brazil’s contribution to building practical, inclusive, and evidence-based solutions, placing health where it always belongs, i.e., at the center of global climate policy.
